# Pericardial effusion with tamponade: bedside ultrasonography saves another life

**DOI:** 10.1186/s12245-019-0257-4

**Published:** 2020-01-28

**Authors:** Javier Rosario, Rohan Mangal, Jessica Houck, Mary Cate Slome, Latha Ganti

**Affiliations:** 1 Envision Physician Services, Nashville, TN USA; 2 UCF HCA Emergency Medicine Residency Program of Greater Orlando, University of Central Florida College of Medicine, Orlando, FL USA; 3 Johns Hopkins University, Baltimore, MD USA

**Keywords:** Pericardial tamponade, Bedside ultrasonography, Acute dyspnea

## Abstract

In these video clinical images, the authors present the cause for an elderly gentleman’s shortness of breath. It was presumed to be an exacerbation of chronic obstructive pulmonary disease, a condition for which he was in the process of being evaluated. However, bedside ultrasonography revealed a large pericardial effusion with tamponade. This timely diagnosis resulted in the patient being taken expeditiously to the operating room and saving his life.

## Introduction

Pericardial effusion describes excessive fluid buildup in the pericardium and is often the cause of infection, renal failure, or hypothyroidism. Indications of pericardial effusion include orthopnea, tachycardia, and shortness of breath; however, clinical suspicion is seldom conclusive—echocardiography is often recommended for confirmation of diagnosis and to provide insight on the patient’s hemodynamic condition [1]. Alternatively, computer tomography (CT) or magnetic resonance imaging (MRI) scan can assist similarly in diagnosis while also uniquely depicting the entire chest and the spatial distribution of pericardial effusion [2]. Ordinarily, the pericardial sac contains up to 50 mL of fluid, with any amount greater indicating a pericardial effusion. When its contents exceed 150 to 200 mL, life-threatening cardiac tamponade may occur [3]. Cardiac tamponade is the heart’s compromised ability to pump due to increased intrapericardial pressure from fluid buildup. Patients with cardiac tamponade may experience chest pain, elevated respiratory rate, shortness of breath, and an upset stomach. However, an echocardiogram may suggest hemodynamic compromise in patients with moderate or severe pericardial effusion who do not display traditional symptoms of tamponade—thereby emphasizing the utility of pericardial imaging modalities [4]. Following diagnosis of cardiac tamponade, emergent drainage of pericardial fluid is strongly recommended to restore hemodynamic functions [5]. The most common cause of pericardial effusion with tamponade is malignant disease [1].

## Case presentation

A 73-year-old man being treated in the outpatient setting for a presumptive diagnosis of chronic obstructive pulmonary disease (COPD) with shortness of breath that was not improving presented to an emergency department (ED) with chief complaints of tachycardia and shortness of breath while in the office. He was sent to the ED after evaluation showed unstable vital signs. Upon physical examination, the patient had a blood pressure of 77/52 mmHg, respiratory rate of 20 breaths per minute, pulse of 102 beats per minute, and a temperature of 96.6 °F. Repeat blood pressure was 66/44 mmHg; however, the patient was experiencing no acute distress and was awake and alert. At this time, the bedside ultrasound machine was brought in to evaluate the cause for hypotension. Sepsis was not yet being considered in the differential.

Bedside ultrasonography revealed a large pericardial effusion with evidence of tamponade. Notable on the video images (Additional files 1 and 2) is a qualitatively large pericardial effusion with clear evidence of tamponade. Interestingly, the only finding on ECG was low voltage; no electrical alternans was appreciated on ECG. Further, only two of the three Beck’s triad elements were met, as the patient presented with both muffled heart sounds and hypotension, but did not have elevated jugular venous distension.


**Additional file 1.** **Video 1.** *Apical four chamber view of the heart (A4C):* Notice this view typically shows the four chambers of the heart including the left ventricle, right ventricle, left atrium, and right atrium. A large pericardial effusion (asterisk) is seen within the pericardium.



**Additional file 2.** **Video 2.** *Parasternal long axis of the heart (PLAX):* This view typically shows four chambers of the heart including the right ventricle, aortic outflow tract, left ventricle, and the left atrium. A large pericardial effusion (asterisk) is seen within the pericardium.


The patient was then transferred to an operating room after brief stabilization in the ED. There, a pericardial window was performed over the xiphoid, in which 1650 mL of bloody fluid was drained. Additionally, a 19 French Blake drain was placed in the pericardial space. Following this drainage of fluid, the patient was hemodynamically stable with an improvement in blood pressure. The patient was discharged home on postoperative day 7 with a diagnosis of malignant pericardial effusion due to metastatic, poorly differentiated lung adenocarcinoma.

## Discussion

Given that 50 mL of fluid delineates the upper limit for normal pericardial volume, this patient presents a severe case of pericardial effusion with 33 times this amount. Pericardiocentesis and a pericardial window are two common techniques for treating large pericardial effusions and cardiac tamponade. Complete fluid evacuation is important to avoid relapse, and thus, pericardiocentesis is sometimes neglected [6]. Within surgical interventions to evacuate the fluid exists the subxiphoid and thoracotomy techniques. While some studies have suggested respiratory complications associated with the thoracotomy approach, both are generally accepted for removing fluid buildup [7].

Point-of-care ultrasound can be used to quickly and noninvasively identify many pathologies, not limited to pericardial effusion, at the bedside. Right heart strain that may suggest pulmonary embolism (or pneumothorax, pulmonary disease, RV infarct), globally reduced ejection fraction, and valvular dysfunction with regurgitation are just some of the pathologies that are easily identifiable at the bedside, during stabilization in the ED [8, 9]. Identification of these abnormalities with employment of bedside ultrasound techniques can expedite lifesaving plans of care for severely ill patients.

Ultrasound examination of the heart, usually via echocardiography, is considered the gold standard for evaluating for pericardial effusion, with or without tamponade. Specific findings include right ventricular diastolic collapse, right atrial collapse, a dilated inferior vena cava, and inspiratory valvular changes. Finally, qualitative observation of the heart swinging within the echolucent space with each beat indicates tamponade [10].

## Conclusion

Swift identification of this patient’s pericardial effusion with tamponade in the emergency department was critical to making an appropriate and timely disposition. This patient was able to be transferred directly to cardiothoracic surgery without the use of advanced imaging, for which the patient was too unstable given the presenting blood pressure, with the use of point-of-care cardiac ultrasound which immediately identified the large effusion with tamponade.

## Data Availability

N/A.

## Abbreviations

COPD: Chronic obstructive pulmonary disease
CT: Computer tomography
ECG: Electrocardiogram
ED: Emergency department
MRI: Magnetic resonance imaging
RV: Right ventricle
